# Effects of Taurine Supplementation on Hepatic Markers of Inflammation and Lipid Metabolism in Mothers and Offspring in the Setting of Maternal Obesity

**DOI:** 10.1371/journal.pone.0076961

**Published:** 2013-10-17

**Authors:** Minglan Li, Clare M. Reynolds, Deborah M. Sloboda, Clint Gray, Mark H. Vickers

**Affiliations:** 1 Liggins Institute and Gravida: National Centre for Growth and Development, University of Auckland, Auckland, New Zealand; 2 Department of Biochemistry and Biomedical Sciences, McMaster University, Hamilton, Ontario, Canada; Monash University, Australia

## Abstract

Maternal obesity is associated with obesity and metabolic disorders in offspring. However, intervention strategies to reverse or ameliorate the effects of maternal obesity on offspring health are limited. Following maternal undernutrition, taurine supplementation can improve outcomes in offspring, possibly via effects on glucose homeostasis and insulin secretion. The effects of taurine in mediating inflammatory processes as a protective mechanism has not been investigated. Further, the efficacy of taurine supplementation in the setting of maternal obesity is not known. Using a model of maternal obesity, we examined the effects of maternal taurine supplementation on outcomes related to inflammation and lipid metabolism in mothers and neonates. Time-mated Wistar rats were randomised to either: 1) control : control diet during pregnancy and lactation (CON); 2) CON supplemented with 1.5% taurine in drinking water (CT); 3) maternal obesogenic diet (high fat, high fructose) during pregnancy and lactation (MO); or 4) MO supplemented with taurine (MOT). Maternal and neonatal weights, plasma cytokines and hepatic gene expression were analysed. A MO diet resulted in maternal hyperinsulinemia and hyperleptinemia and increased plasma glucose, glutamate and TNF-α concentrations. Taurine normalised maternal plasma TNF-α and glutamate concentrations in MOT animals. Both MO and MOT mothers displayed evidence of fatty liver accompanied by alterations in key markers of hepatic lipid metabolism. MO neonates displayed a pro-inflammatory hepatic profile which was partially rescued in MOT offspring. Conversely, a pro-inflammatory phenotype was observed in MOT mothers suggesting a possible maternal trade-off to protect the neonate. Despite protective effects of taurine in MOT offspring, neonatal mortality was increased in CT neonates, indicating possible adverse effects of taurine in the setting of normal pregnancy. These data suggest that maternal taurine supplementation may ameliorate the adverse effects observed in offspring following a maternal obesogenic diet but these effects are dependent upon prior maternal nutritional background.

## Introduction

Obesity and overweight during pregnancy has become a major emerging issue for maternal and neonatal health over the past decade [1], [2]. Periconceptional and gestational obesity are associated with insulin resistance (IR) and low-grade inflammation which increases the incidence of gestational diabetes, preeclampsia, miscarriage, and neonatal mortality and the long-term risk of developing metabolic syndrome [3]–[5]. A recent clinical study highlighted the relationship between intrahepatic fat and IR in women with previous gestational diabetes (GDM) [6], indicating mild hepatic steatosis in postpartum women may contribute to IR-related metabolic dysfunction.

In addition to metabolic disorders and adverse pregnancy outcomes, maternal obesity has been shown to impact the long term health of the offspring [7]. The developmental origins of health and disease (DOHaD) paradigm proposes that insults such as poor maternal nutrition during critical windows of development, can lead to an increased propensity in offspring to develop obesity and related metabolic and cardiovascular disorders in later life [8]. Both human studies [9], [10] and animal models [11], [12] clearly show a link between maternal obesity and heightened risk of metabolic disorders in offspring, yet relatively little is known about the mechanisms involved. Therefore, broad lifestyle recommendations remain the most common preventative strategies [7].

A number of studies have reported the effectiveness of taurine (2-aminoethanesulfonic acid) in treating IR [13]–[15]. Taurine is a sulphonic amino acid derived from methionine and cysteine metabolism and is found ubiquitously in all mammalian tissues. The synthesis and metabolism of taurine has known species-specific differences although taurine can be synthesised *in vivo* in both the human and rodent [16]. Taurine is involved in bile acid synthesis, osmoregulation, modulation of neurotransmitters, glucose homeostasis and insulin secretion [17], [18]. Reports suggest that taurine supplementation can enhance insulin sensitivity through modification of insulin signaling enzymes in fructose-fed rats [19]. Furthermore, maternal taurine supplementation to low protein mothers has been documented to normalise pancreatic islet development in offspring with normalisation of glucose and insulin homeostasis in later life [20]–[22]. These beneficial effects on glucose metabolism have been shown to persist into adult life [23]. Although the effects of maternal taurine supplementation as relates to improved glucose homeostasis and beta-cell function in offspring have been well documented, the direct effects of taurine supplementation on the mother are not well documented. Further, taurine has been proposed to play a role in mediating inflammatory processes but this has yet to be examined as a potential mechanism by which maternal taurine supplementation leads to protective effects in the offspring. Recent work by Lin *et al.* has shown that taurine can improves obesity-induced inflammatory responses and modulates the unbalanced phenotype of adipose tissue macrophages [24]. Obesity is characterised by a state of low grade inflammation and maternal obesity is well established to lead to obesity and related metabolic disorders in offspring [12], [25]. In this context, the efficacy of maternal taurine supplementation as an intervention in the setting of maternal obesity has yet to be investigated. Since most studies in the area of developmental programming focus on offspring outcomes, very little attention is paid to the direct effects on maternal health and wellbeing. The current study therefore investigated the effect of taurine supplementation to pregnant and lactating dams fed either a control or obesogenic diet on both maternal and offspring metabolic and hepatic inflammatory profiles.

## Methods

### Animal Model

#### Ethics statement

All procedures described were approved by the Animal Ethics Committee at the University of Auckland (Approval R888).

Virgin Wistar rats were time mated at 100 days of age using an estrous cycle monitor (EC-40, Fine Science Tools, San Francisco, USA). Day 1 of pregnancy was determined by the presence of spermatozoa after a vaginal smear. Pregnant rats were then housed individually with free access to food and water and maintained at 25°C and a 12 h light: 12 h darkness cycle. Animals were randomly assigned to one of four nutritional groups: control group (CON) fed a standard chow diet (Diet 2018, 24% calories from protein, 18% from fat, 58% from carbohydrate, Harlan Teklad, Blackthorn, Bicester, UK) *ad-libitum* throughout pregnancy and lactation (n = 9); control taurine group (CT) fed standard chow diet with additional 1.5% w/v taurine supplementation in drinking water [20], [22] (n = 7); maternal obesogenic diet group (MO) fed a high-fat high-fructose diet (20% calories from protein, 45% from fat, 35% from carbohydrate (including 26% from fructose); Diet D03101602, Research Diets, NJ, USA; n = 8); maternal obesogenic diet and taurine group (MOT) fed the obesogenic diet with additional 1.5% w/v taurine supplementation in drinking water (n = 8).

Two discrete time points were investigated. Firstly, effects of maternal taurine supplementation on neonatal outcomes and secondly the direct effects of taurine on maternal lipid and inflammatory profiles at the end of the lactation period. Maternal body weight, food and fluid intake were recorded daily. After birth, litter size was adjusted to 8 pups per litter (post-weaning offspring were utilised in an independent study). Neonatal plasma and liver samples were collected from randomly chosen excluded pups following decapitation. Litter size, sex ratio and birth weight were recorded at the time of birth. At the end of lactation, dams were fasted overnight and killed by decapitation following anaesthesia with sodium pentobarbitone (60 mg/kg IP). Maternal body composition was measured by dual energy X-ray absorptiometry using dedicated small animal software (DEXA, Lunar Prodigy, Madison, WI, USA). Maternal and neonatal blood glucose and β-hydroxybutyrate (BHB) were measured from tail blood samples using a glucose meter (Optium, Abbott Laboratories) at the time of cull.

### Plasma Analysis

ELISA kits were used to measure plasma insulin and leptin (CrystalChem, USA), tumour necrosis factor (TNF)-α, interleukin (IL)-1β and IL-6 (Quantikine ELISA; R&D Systems Europe, Abingdon, UK). Plasma uric acid was measured using a commercially sourced assay kit (Cayman Chemical, Ann Arbor, MI, USA). The homeostasis model assessment of insulin resistance (HOMA-IR) was calculated as: Fasting glucose (mmol/l)×fasting insulin (mU/l)/22.5 [26]. Maternal plasma glutamate and taurine were analysed using a Hitachi 902 autoanalyser (Hitachi High Technologies Corporation, Tokyo, Japan). Homocysteine (HcY) was measured by commercial immunoassay (Abbott AxSYM system). Limited plasma sample precluded measurement of taurine, glutamate or HcY in neonatal samples.

### Hepatic mRNA Expression

Total RNA was isolated from liver tissue using RNeasy® mini kit (QIAGEN, Hilden, Germany) and cDNA synthesized from 2 µg of RNA by using SuperScript® VILO™ cDNA Synthesis Kit (Invitrogen™; Life Technologies Corporation, California, USA). Real-time PCR analysis for maternal hepatic sterol regulatory element-binding protein-1c (SREBP-1c), peroxisome proliferator-activated receptor alpha (PPARα), lipoprotein lipase (LPL), silent mating type information regulation 2 homolog 1 (SIRT1), fructokinase and phosphoenolpyruvate carboxykinase (PEPCK) expression was performed using LightCycler®480 SYBR green I master (Roche Diagnostics; Auckland, New Zealand). The relative amounts of genes were quantitated using standard curve and normalized to the geometric mean of cyclophilin A and β-actin expression. Real time PCR analysis for maternal hepatic fatty acid synthase (FASN), CD36, TNFα, IL-1β, IL-1R1 and all neonatal samples was carried out by using PreDeveloped TaqMan® Assay Reagent Kits in the ABI 7900HT Fast Real-Time PCR System (Applied Biosystems). To control for between-sample variability, mRNA levels were normalized to the geometric mean of cyclophilinA and hypoxanthine phosphoribosyltransferase (HPRT) for each sample by subtracting the Ct of controls from the Ct for the gene of interest producing a Ct value. The ΔCt for each treatment sample was compared to the mean ΔCt for control samples using the relative quantification 2-(ΔΔCt) method to determine fold-change [27]. Primer details are provided in Table S1.

### Histological Analysis

5 mm thick representative sections from the left lobe of maternal livers were fixed in paraformaldehyde and paraffin embedded. Cross sections were prepared using Leica RM 2135 rotary microtome (Leica Instruments, Nussloch, Germany). Haematoxylin and Eosin (H&E) staining was conducted for general histology. Sections were mounted using distrene plasticizer xylene (DPX) mounting medium (BioLab ltd, New Zealand) and analysed under light microscope (Nikon 800, Tokyo, Japan) and images taken (Nikon FDX-35, Tokyo, Japan) and processed with NIS Elements-D software (Nikon, Tokyo, Japan). Scoring of steatosis, lobular inflammation, hepatocyte ballooning and overall NAS score (NAFLD Activity Score) was undertaken by a blinded observer using the methodology of Kleiner *et al*. [28]. The scoring system comprised four primary features evaluated semi-quantitatively: steatosis (0–3), lobular inflammation (0–3), hepatocellular ballooning (0–2) and NAS score (unweighted sum of steatosis, lobular inflammation, and hepatocellular ballooning scores).

### Statistical Analysis

Data analysis was completed using factorial analysis of variance (ANOVA) using SigmaStat software (Systat Software, San Jose, Ca, USA). For the maternal data, maternal diet and taurine were used as factors. For neonatal data, maternal diet, taurine and were used as factors. Where appropriate, post-hoc analyses were performed (Holm-Sidak method) to determine which groups were significantly different from each other. Maternal body weight data was analysed using repeated measures analysis. Maternal liver histology data were analysed via non-parametric methods (Wilcoxon rank sum test followed by Bonferonni correction). Data that failed to meet the criteria required for parametric analysis (normal distribution and equal variance) were transformed where necessary. All data are shown as means ± SEM. A p-value of <0.05 was accepted as statistically significant.

## Results

### Maternal and Offspring Weights

**Figure 1:**
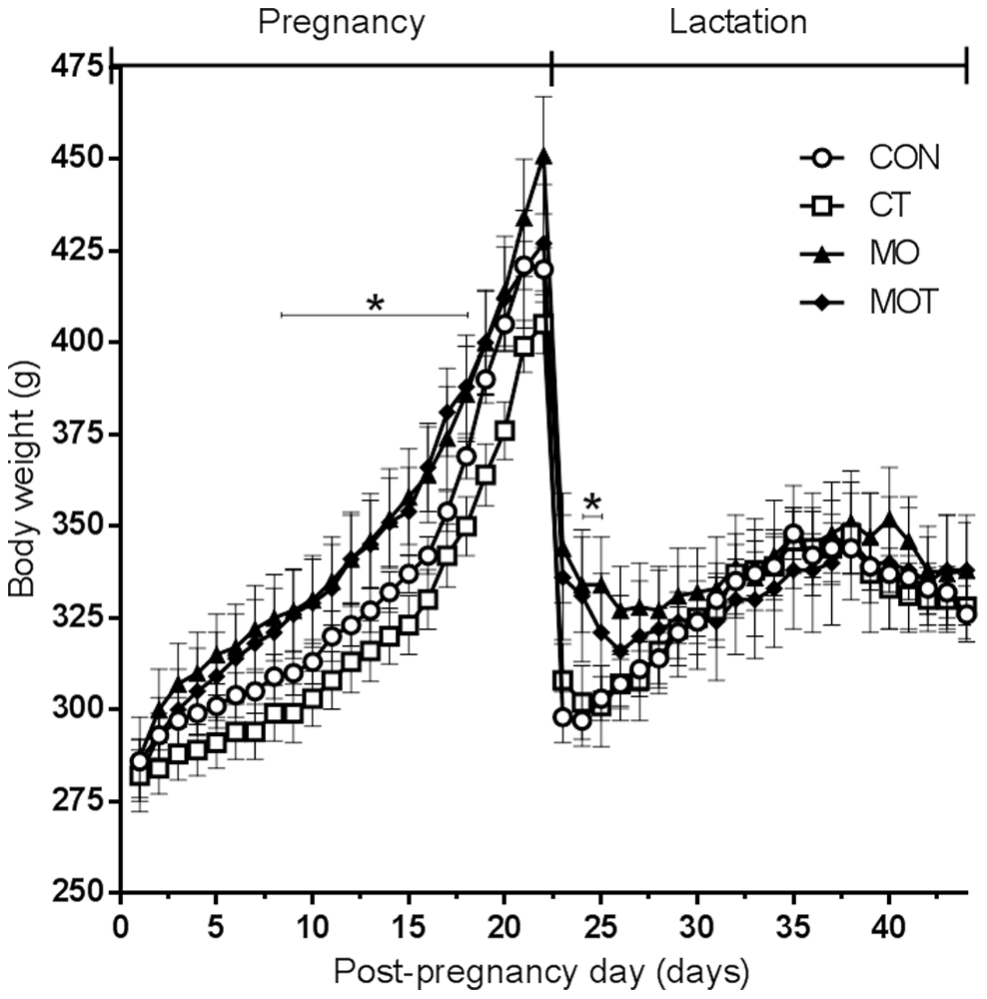
Maternal body weight during pregnancy and lactation. Body weights were measured daily throughout pregnancy and gestation. Data are means ± SEM, n = 7–9 per group.

A maternal obesogenic diet resulted in an overall increase in maternal body weights in both MO and MOT groups (Figure 1) during pregnancy compared to CON and CT groups and was statistically significant from day 8 to day 18 gestation. Of note there was a small decrease in maternal body weights in CT dams compared to CON which was not evident in the MOT group and this was reflected in a significant maternal diet x taurine statistical interaction (p<0.0001). In the immediate post-partum period, MO and MOT dams remained significantly heavier than CON and CT mothers but there were no differences in maternal body weights between groups for the remainder of the lactation period (Figure 1). Birth weights were significantly reduced in female MO and MOT offspring compared to CON and CT groups (Table 1) but were not different between male groups. Interestingly, maternal taurine supplementation significantly increased neonatal mortality in the CT group (CON 1.4±0.1%; CT 7.1±4.0%, p<0.05), without significant difference between CON, MO and MOT groups. Litter size and sex ratios were not affected by maternal diet or taurine supplementation (data not shown). Total maternal fat mass was significantly increased in MO but not in MOT animals when compared with CON at the end of lactation (Table 1). An effect of maternal diet was observed on relative maternal liver weight, increasing significantly in MO and MOT groups compared to CON (Table 1). Weaning weight was significantly increased in MO offspring compared to CON. There was a significant maternal diet x taurine interaction (p = 0.01) in males whereby CT body weights were higher than CON but the reverse holding true in MO groups with MO weights being higher than MOT. Weaning (postnatal day 22) weights were not significantly different between any of the female offspring groups (Table 1).

**Table 1:** Maternal, neonatal and weaning weight data.

	Groups				Effect		
	CON	CT	MO	MOT	Diet	Taurine	Interaction
Maternal weight (g)	297.8±6.6^B^	298.7±8^B^	335.3±13.9^A^	330.3±15.9^A^	**F = 8.461** **P = 0.007**	F = 0.126P = 0.726	F = 0.00835P = 0.928
Maternal liver weight(% body weight)	3.78±0.1^B^	3.79±0.1^B^	5.16±0.3^A^	5.16±0.2^A^	**F = 53.203** **P<0.001**	F = 0.000168P = 0.990	F = 0.00149P = 0.969
Maternal total fat (%)	10.7±1.7^B^	13.8±2.7^B^	21.1±4. 8^A^	15.9±1.7^A^	**F = 6.140** **P = 0.020**	F = 0.0465P = 0.831	F = 1.118P = 0.300
Birthweights (male, g)	6.34±0.1	5.80±0.1	5.96±0.2	5.89±0.2	F = 0.895P = 0.348	F = 3.902P = 0.053	F = 2.410P = 0.126
Birthweights (female, g)	6.32±0.1^A^	5.94±0.2^A^	5.36±0.2^B^	5.41±0.1^B^	**F = 29.175** **P = 0.003**	F = 0.481P = 0.491	F = 1.015P = 0.318
Weaning weight (male, g)	59.9±0.7^b^	62.0±1.2^b^	64.5±1.9^a^	60.7±1.2^b^	F = 1.804P = 0.182	F = 0.451P = 0.503	**F = 5.612** **P = 0.019**
Weaning weight (female, g)	57.8±0.9	60.3±1.3	59.2±1.1	58.7±0.9	F = 0.004P = 0.947	F = 0.933P = 0.336	F = 2.176P = 0.143

Values are presented as means ± SEM, n = 7–9 per group for maternal data, minimum 20 per group for birth and weaning weights. Bold font indicates effect P value <0.05 via two-way ANOVA. Upper case letter (^A^,^B^) superscripts indicate comparison procedures were conducted between all groups fed MO diet and all groups fed CON diet. Lower case letter superscripts (^a,b^) indicate multiple comparison procedures were conducted for diet and taurine interaction. Groups that do not share the same letter are significantly different from each other (p<0.05).

### Maternal Plasma Profile

**Figure 2:**
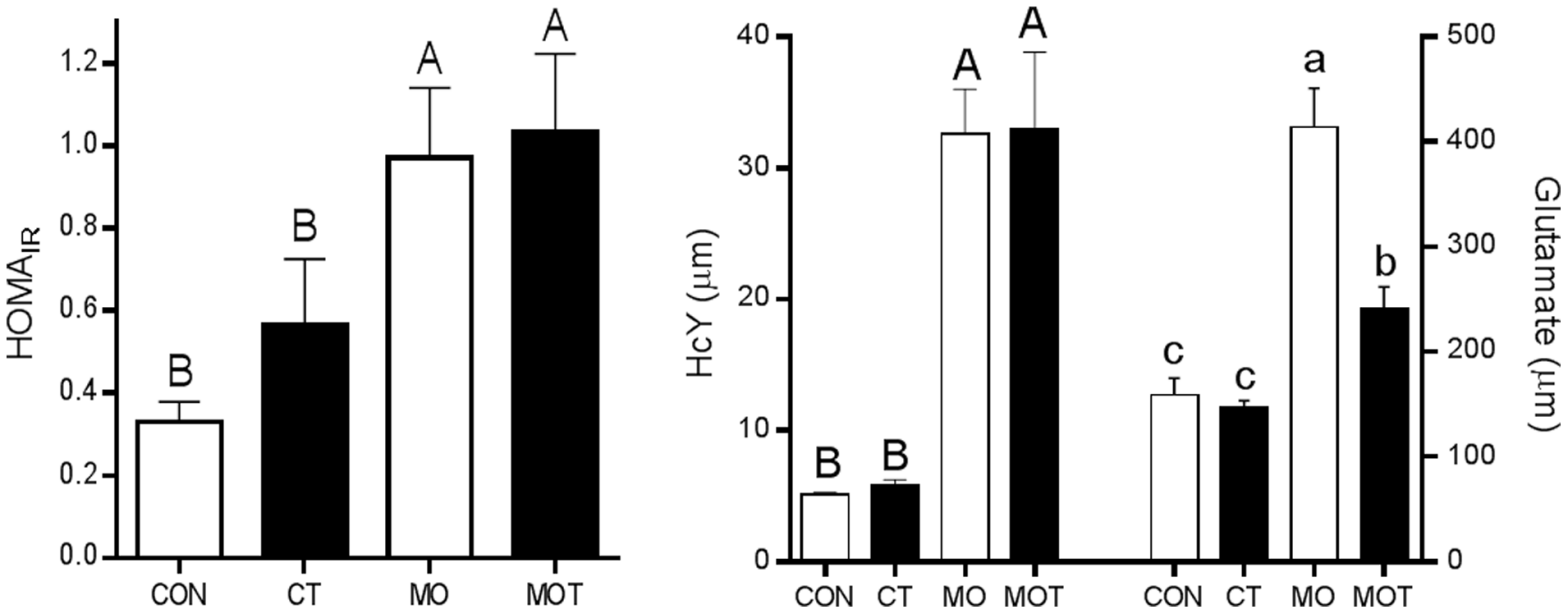
(a) Maternal HOMA_IR_ values. Data are means ± SEM, n = 7–9 per group. (b) Maternal plasma homocysteine (HcY) and glutamate concentrations. Data are means SEM, n = 7–9 per group. Upper case letter (^A^, ^B^) superscripts indicate comparison procedures were conducted between all groups fed MO diet and all groups fed CON diet. Lower case letter superscripts (^a,b,c^) indicate multiple comparison procedures were conducted for diet and taurine interaction. Groups that do not share the same letter are significantly different from each other (p<0.05).

Maternal plasma taurine was increased in CT and MOT groups compared to CON and MO groups (Table 2). Maternal fasting glucose was increased while BHB concentrations decreased in MO and MOT groups compared to CON and CT groups and there was no effect of taurine (Table 2). Maternal plasma uric acid was significantly decreased in MOT compared to MO groups (Table 2). MO and MOT groups displayed significant hyperinsulinemia and increased HOMA-IR indices when compared to CON and CT groups (Table 2 and Figure 2a). Hyperleptinemia was observed in MO but not MOT groups although there was no significant overall effect of taurine supplementation. TNF-α was significantly increased in MO compared to CON, CT and MOT groups. Plasma IL-1β and IL-6 concentrations were not affected by diet or taurine supplementation (Table 2). Maternal plasma homocysteine (HcY) concentrations were significantly increased in response to the maternal obesogenic diet in MO and MOT dams (Figure 2b). Plasma glutamate concentrations were significantly increased in MO dams compared to all other groups (Figure 2b, maternal diet x taurine interaction p<0.05).

**Table 2:** Maternal plasma profile.

	Groups				Effect		
	CON	CT	MO	MOT	Diet	Taurine	Interaction
Taurine (umol/l)	328±25^B1^	436±40^A1^	341±20^B1^	415±32^A1^	F = 0.00304P = 0.956	**F = 10.186** **P = 0.004**	F = 0.397P = 0.534
Glucose (mmol/l)	6.11±0.49^B^	7.0±0.40^B^	8.2±0.56^A^	7.79±0.30^A^	**F = 9.599** **P = 0.005**	F = 0.450P = 0.508	F = 2.371P = 0.136
BHB (mmol/l)	1.09±0.12^A^	0.91±0.11^A^	0.73±0.09^B^	0.64±0.04^B^	**F = 8.705** **P = 0.007**	F = 1.478P = 0.235	F = 0.172P = 0.681
Uric acid (µM)	2.983±0.454	3.797±0.593	2.636±0.812	1.845±0.301	F = 3.955P = 0.057	F = 0.113P = 0.739	F = 1.083P = 0.308
Leptin (ng/ml)	1.420±0.129^B^	1.611±0.176^B^	1.994±0.161^A^	1.671±0.150^ A^	**F = 4.249** **P = 0.049**	F = 0.183P = 0.672	F = 2.801P = 0.106
Insulin (ng/ml)	1.247±0.139^B^	1.728±0.381^B^	2.665±0.402^A^	2.980±0.564^A^	**F = 17.114** **P<0.001**	F = 1.027P = 0.320	F = 0.427P = 0.519
TNFα (µg/l)	0.111±0.008^b^	0.129±0.013^ab^	0.155±0.017^a^	0.102±0.006^b^	F = 0.140P = 0.711	F = 4.420P = 0.045	**F = 10.860** **P = 0.003**
IL1β (µg/l)	0.116±0.006	0.118±0.005	0.118±0.006	0.102±0.003	F = 1.539P = 0.226	F = 1.815P = 0.190	F = 2.753P = 0.109
IL6 (µg/l)	0.172±0.013	0.165±0.016	0.141±0.017	0.185±0.019	F = 0.152P = 0.700	F = 1.589P = 0.219	F = 2.300P = 0.141

Values are presented as mean ± SEM, n = 7–9 per group. Bold font indicates effect P value <0.05 via two-way ANOVA, main effects were not indicated when a significant interaction was determined. Comparisons between groups for each significant effect where applicable are denoted by 3 sets of superscripts. Upper case letter (^A^, ^B^) superscripts indicate comparison procedures were conducted between all groups fed MO diet and all groups fed CON diet. Upper case letter (^A1^, ^B1^) superscripts indicate comparison procedures were conducted between groups with taurine supplementation and groups without taurine supplementation. Lower case letter superscripts (^a,b,c^) indicate multiple comparison procedures were conducted for diet and taurine interaction. Groups that do not share the same letter are significantly different from each other (p<0.05).

### Maternal Hepatic Morphology

**Figure 3:**
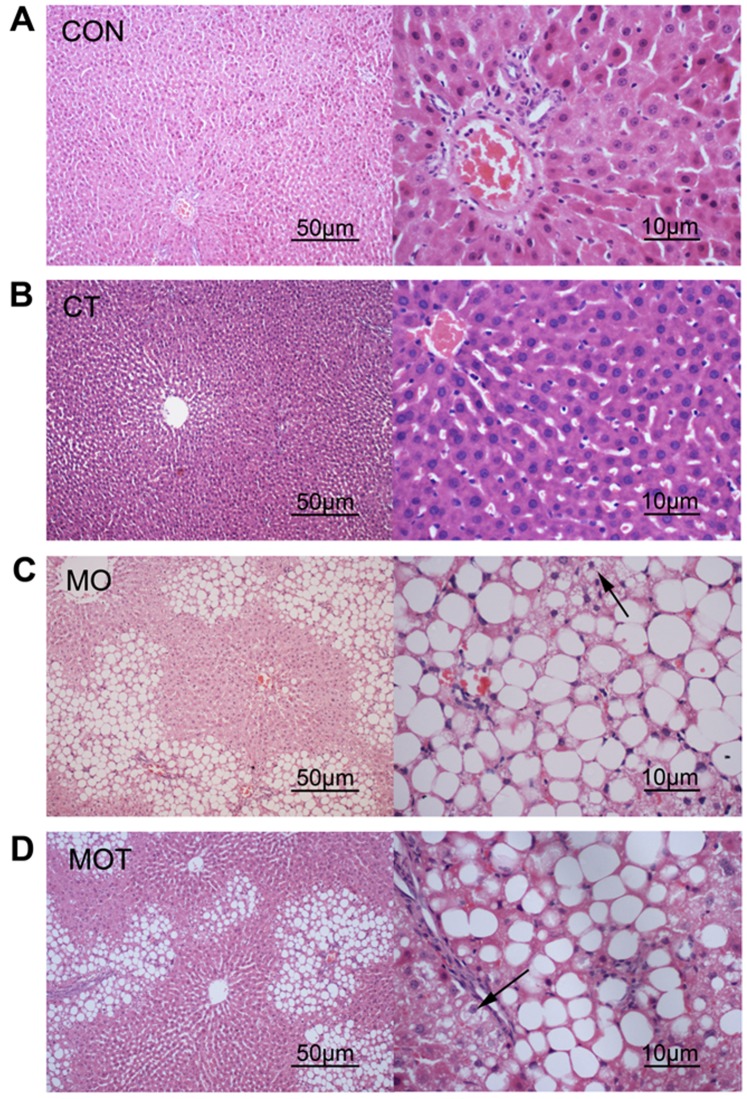
Maternal liver histology. Left column, H&E, 10× magnification; right column 40× magnification. CON: control, n = 9; CT: control with taurine, n = 7; MO: maternal obesogenic diet, n = 8; MOT: MO maternal obesogenic diet with taurine, n = 7. Arrows indicate ballooned hepatocytes.

Given the changes observed in maternal liver weights in response to diet as shown in Table 1, analysis of the histological features associated with NAFLD was undertaken using the method of Kleiner *et al*. [28]. Scores related to steatosis, lobular inflammation, hepatocyte ballooning and overall NAS score were significantly increased in MO and MOT groups compared to CON and CT groups. There was no significant effect of maternal taurine supplementation on any of the markers analysed (Table 3 and Figure 3).

**Table 3:** Maternal liver scoring.

	Groups			
	CON	CT	MO	MOT
Steatosis	0±0^b^	0±0^b^	1.38±0.18^a^	1.29±0.18^a^
Lobular inflammation	0.3±0.15^b^	0±0^b^	1.88±0.30^a^	1.43±0.20^a^
Hepatocyte ballooning	0±0^b^	0±0^b^	1.38±0.18^a^	1.14±0.14^a^
NAS score	0.3±0.15^b^	0±0^b^	4.63±0.53^a^	3.86±0.34^a^

Values are presented as means ± SEM, n = 7–9 per group. Pairwise comparisons between groups were conducted via Wilcoxon rank sum test with Bonferroni correction, groups that did not share the same letter are significantly different from each other (p<0.05).

### Maternal Hepatic Gene Expression

**Figure 4:**
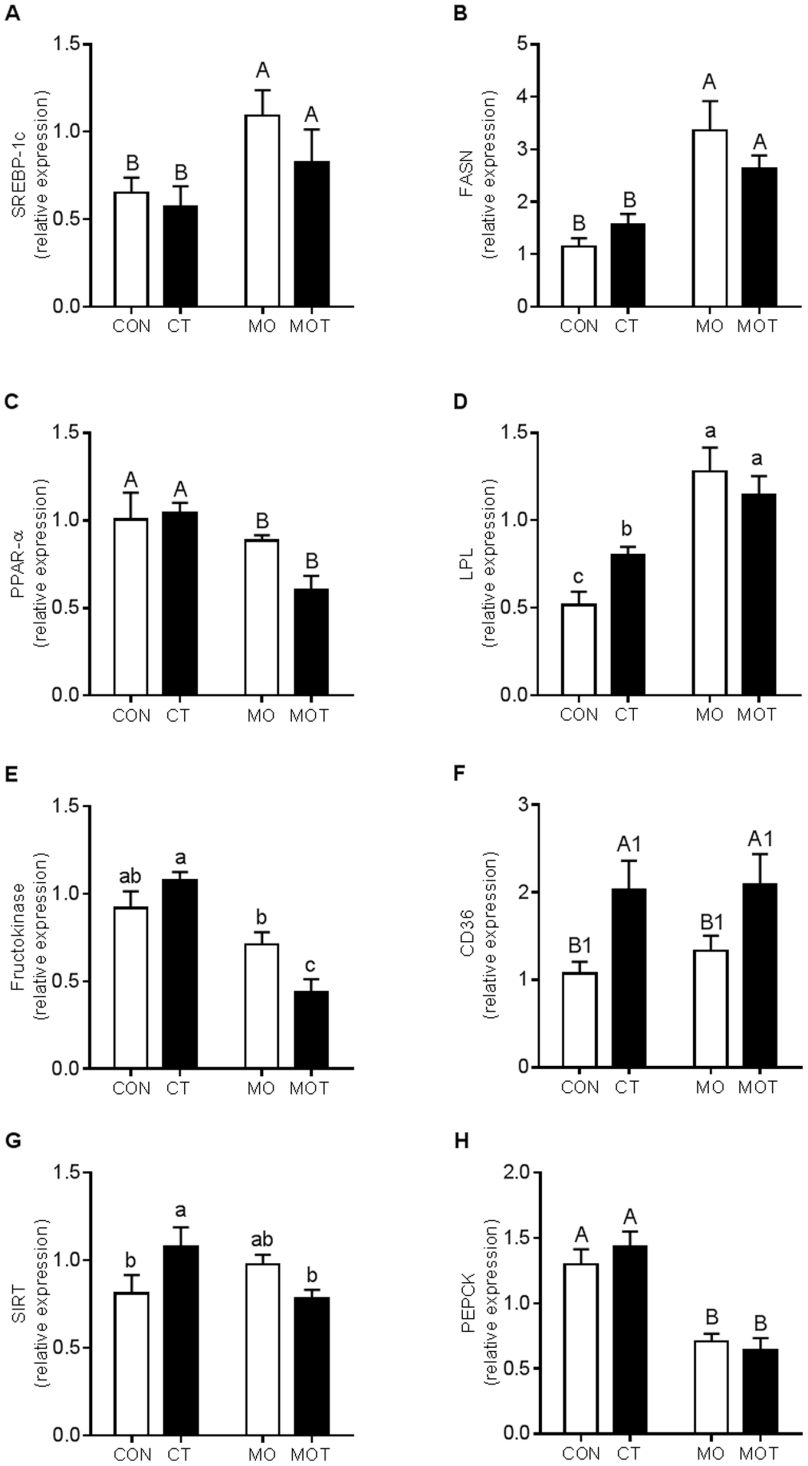
Maternal hepatic lipid and glucose metabolism related gene expression. Values are presented as mean ± SEM, n = 7–9 per group. Bold font indicates effect P value <0.05 via two-way ANOVA, main effects were not indicated when significant interaction was determined. Comparisons between groups for each effect where significant are denoted by 3 sets of superscripts. Upper case letter (^A^, ^B^) superscripts indicate comparison procedures were conducted between all groups fed MO diet and all groups fed CON diet. Upper case letter (^A1^, ^B1^) superscripts indicate comparison procedures were conducted between groups with taurine supplementation and groups without taurine supplementation. Lower case letter superscripts (^a,b,c^) indicate multiple comparison procedures were conducted for diet and taurine interaction. Groups that do not share the same letter are significantly different from each other (p<0.05).

**Figure 5:**
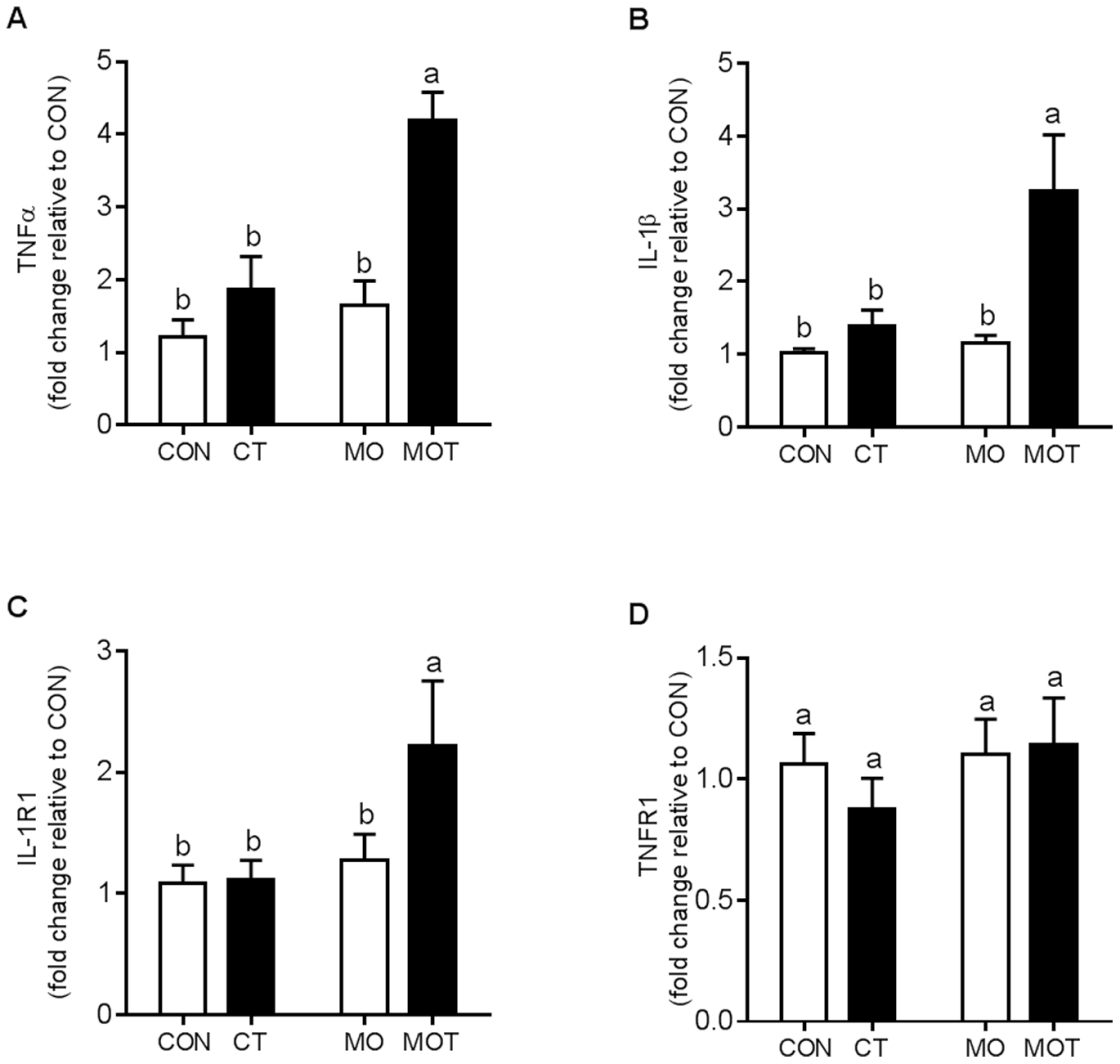
Maternal hepatic inflammatory gene expression. (a) TNFα; (b) IL-1β; (c) IL-1R1; (d) TNFR1. Lower case letter superscripts (^a,b,c^) indicate multiple comparison procedures were conducted for diet and taurine interaction. Groups that do not share the same letter are significantly different from each other (p<0.05). Data are means ± SEM, n = 7–9 per group.

To further assess the fatty liver phenotype observed via hepatic morphology, we conducted gene expression analysis for genes related to lipogenesis and inflammatory responsiveness. Hepatic SREBP-1c expression was significantly increased in the MO and MOT groups when compared to the CON and CT groups (Figure 4a). FASN expression was increased in both MO and MOT groups compared to CON and CT (Figure 4b). PPARα was significantly down-regulated in MO and MOT groups compared to CON and CT groups (Figure 4c). LPL was significantly increased in MO and MOT groups compared to CON and CT and increased in CT versus CON groups (Figure 4d). Fructokinase expression was significantly reduced in MOT group compared to CON, CT and MO groups (Figure 4e). CD36 expression was significantly increased in CT and MOT groups when compared to CON and MO groups (Figure 4f). SIRT1 expression was increased in CT versus CON and MOT groups (Figure 4g). PEPCK was significantly reduced in both MO and MOT groups compared to CON and CT (Figure 4h). Hepatic TNF-α, IL-1β and IL-1R1 expression was significantly increased in MOT animals compared to CON, CT and MO groups (Figures 5a–c). No effect was observed on maternal hepatic TNFR1 expression (Figure 5d). Overall main effect statistical data are provided in Table S2.

### Neonatal Plasma and Physiology Profile

There were no significant effects of maternal diet or taurine supplementation on neonatal plasma insulin or leptin concentrations although there was a trend toward reduced leptin concentrations in MO and MOT offspring versus controls (p = 0.075 and p = 0.07 for males and females respectively, Table 4). Plasma BHB concentrations were significantly reduced in CT, MO and MOT offspring compared to CON and there was a significant diet and taurine interaction in both male and female neonates (CON>CT but MO<MOT) (Table 4). Female offspring relative liver weights were significantly increased in MO and MOT compared to CON and CT groups (CON 3.9±0.1%; CT 3.8±0.2%; MO 4.0±0.1%; MOT 4.2±0.1%). Interestingly, male offspring relative liver weights did not differ among groups (data not shown).

**Table 4:** Neonatal plasma profile.

	Groups				Main Effect		
	CON	CT	MO	MOT	Diet	Taurine	Interaction
**Females**							
Leptin (ng/ml)	1.505±0.304	1.147±0.440	0.763±0.277	0.816±0.221	F = 3.477P = 0.075	F = 1.720P = 0.203	F = 1.709P = 0.204
Insulin (ng/ml)	1.81±0.47	0.93±0.25	1.08±0.37	0.86±0.23	F = 1.082P = 0.309	F = 1.980P = 0.172	F = 0.744P = 0.397
Glucose (mmol/l)	4.413±0.176	3.854±0.396	4.493±0.383	4.433±0.290	F = 1.176 P = 0.282	F = 1.032P = 0.313	F = 0.670P = 0.416
BHB (mmol/l)	2.15±0.128^a^	1.455±0.225^b^	1.693±0.174^b^	1.733±0.117^b^	F = 0.322P = 0.572	F = 4.370P = 0.040	**F = 5.502** **P = 0.022**
**Males**							
Leptin (ng/ml)	1.182±0.242	0.527±0.219	0.449±0.112	0.469±0.129	F = 3.618P = 0.070	F = 2.337P = 0.140	F = 2.631P = 0.118
Insulin ( ng/ml)	0.70±0.22	0.54±0.25	0.70±0.26	0.45±0.16	F = 0.291P = 0.595	F = 0.685P = 0.416	F = 0.0372 P = 0.849
Glucose ( mmol/l)	4.684±0.214	5.043±0.244	4.994±0.128	4.581±0.230	F = 0.094P = 0.760	F = 0.012P = 0.912	F = 2.449P = 0.122
BHB ( mmol/l)	2.052±0.082^a^	1.414±0.063^bc^	1.322±0.080^c^	1.757±0.106^b^	F = 3.237P = 0.076	F = 0.889P = 0.349	**F = 24.877** **P<0.001**

Values are presented as mean ± SEM, n = 7–9 per group. Bold font indicates effect P value <0.05 via two-way ANOVA, main effects were not indicated when a significant interaction was determined. Lower case letter superscripts (^a,b,c^) indicate multiple comparison procedures were conducted for diet and taurine interaction. Groups that do not share the same letter are significantly different from each other (p<0.05).

### Neonatal Hepatic Inflammatory Profile

**Figure 6:**
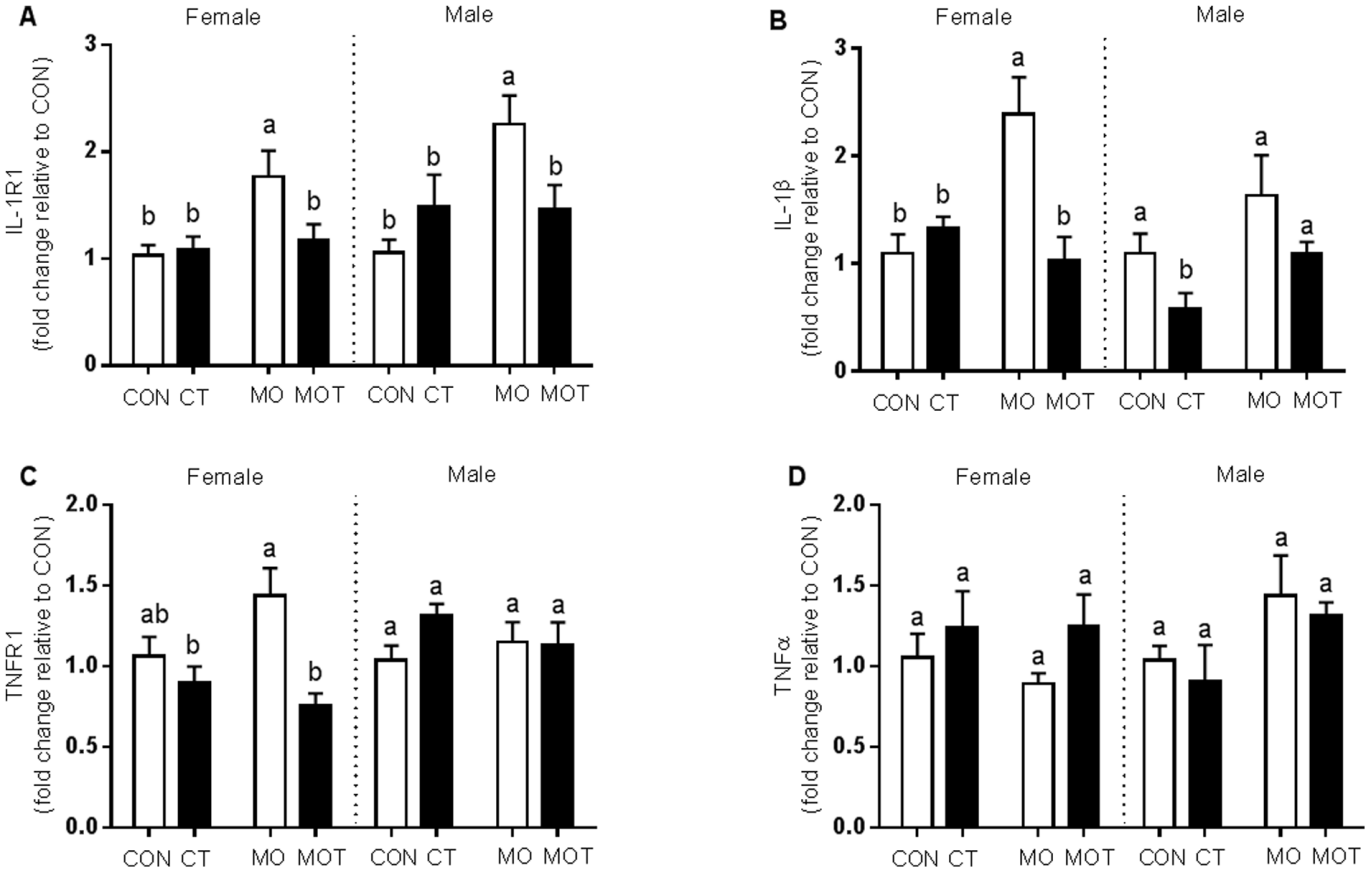
Neonatal liver inflammatory gene expression. Neonatal hepatic gene expression of (a) IL-1R1; (b) IL-1β; (c) TNFR1 and (d) TNF-α. Lower case letter superscripts (^a,b,c^) indicate multiple comparison procedures were conducted for diet and taurine interaction. Groups that do not share the same letter are significantly different from each other (p<0.05). Data are means ± SEM, n = 7–9 per group.

IL-1R1 expression in male and female neonates was significantly increased in MO offspring compared to all other groups (Figure 6a). Hepatic IL-1β expression was increased in female MO neonates compared to all other female groups. In male neonates, IL-1β was decreased in CT versus all other groups (Figure 6b). TNFR1 expression was decreased in MOT female neonates compared to the MO group (Figure 6c). There were no significant differences in TNFR1 between the male neonatal groups. There were no differences in neonatal hepatic TNF-α expression (Figure 6d). There were no significant differences in markers related to IR or lipid metabolism (Figure S1).

## Discussion

The present study demonstrated significant metabolic and inflammatory changes in dams and neonates in response to a maternal high-fat:high-fructose diet. Dams fed the obesogenic diet displayed an adverse metabolic phenotype which included increased weight gain, hyperglycaemia, hyperhomocysteinemia, IR, and evidence of hepatic steatosis and inflammation. Maternal taurine supplementation significantly attenuated the proinflammatory plasma profile and plasma glutamate induced by obesogenic diet. Despite these beneficial systemic effects, maternal hepatic lipid metabolism and inflammatory profile were further impaired in response to taurine supplementation. Conversely, the neonatal hepatic immunophenotype, which was worsened by maternal obesogenic diet, was normalised by maternal taurine supplementation. However, it must be noted that the neonatal and maternal time-points represent independent observations and causative associations can only be speculated upon. Nonetheless, even as independent observations, both the maternal and neonatal data point to a marked effect of taurine supplementation on modifying maternal and neonatal outcomes in the setting of a maternal obesogenic diet.

This study demonstrated that maternal obesogenic diet induced significant hepatic steatosis in dams. Hepatic lipogenic gene expression was altered by the obesogenic diet. Increased SREBP-1c, FASN, LPL and decreased PPARα expression was observed in MO dams. There are three possible main pathways by which these altered gene expression may contribute to hepatic steatosis. Firstly, SREBP-1c is an important transcriptional regulator of fatty acid synthesis and regulates the expression of key enzymes such as acetyl coenzyme-A carboxylase-1 (ACC-1), Stearoyl-CoA desaturase-1 (SCD1) and FASN [29], [30]. SREBP-1c expression is insulin sensitive [31], and overexpression can induce *de novo* lipogenesis via upregulation of FASN [29], [32] potentially contributing to lipotoxicity. Secondly, PPARα is a mediator of hepatic fatty acid β-oxidation [33]. As a member of the nuclear hormone receptor superfamily, PPARα can be attenuated by insulin in hepatocytes [34]. In our model, elevated circulating insulin may contribute to the downregulation of hepatic PPARα and associated reduction in BHB levels indicating reduced fatty acid utilization, a hypothesis supported by others [35]. Thirdly, upregulated LPL expression may contribute to hepatic steatosis by increasing intracellular free fatty acid accumulation through hydrolysis of lipoprotein triglyceride [36], [37]. Interestingly, it has been shown that fructose can induce hepatic steatosis via fructokinase-mediated fructose phosphorylation [38], [39]. However, in the current study fructokinase was downregulated in high-fat high-fructose diet group, suggesting high-fat, rather than high-fructose component of the maternal diet represented the predominant contributor to the maternal hepatic steatosis.

Contrary to obesity studies in non-pregnant animals [40]–[42], maternal diet-induced hepatic steatosis was not reversed, but further aggravated by taurine supplementation. The steatosis observed in the present study represents an instance of non-alcoholic fatty liver disease (NAFLD) which is a chronic condition which is characterized by two distinct phases [43]. Firstly, hyperglycemia and IR contribute to ectopic fat deposition in the liver contributing to intracellular lipid accumulation in hepatocytes increasing oxidative stress and proinflammatory cytokines production which initiate the second phase characterized by hepatocyte apoptosis and progressive fibrosis. In the current study, exacerbated hepatic steatosis in MOT group was evidenced by an elevated hepatic immunophenotype which included upregulated TNF-α and IL-1β expression. It is well established, that TNF-α and IL-1β expression is correlated to the severity of steatohepatitis [44]–[46]. Increasing free fatty acid accumulation promotes this proinflammatory phenotype through activation of the TLR4 signaling pathway which culminates in NF-κB activation [47]. In our study, in response to taurine supplementation, maternal hepatic CD36 expression was markedly increased. CD36 is a long chain fatty acid transporter [48] which can directly bind free fatty acid from plasma [37]. Increased fatty acid uptake via CD36, together with potential de novo lipogenesis resulting from increased FASN expression in MOT mothers may contribute to intracellular hepatic fatty acid accumulation. Therefore, this ectopic fatty acid deposition may exacerbate inflammatory processes triggering NAFLD in taurine supplemented high-fat fed mothers.

Although hepatic proinflammatory profile deteriorated in the MOT group, circulating TNF-α was significantly reduced. Maternal taurine supplementation has been previously shown to down-regulate systemic inflammation in acute trauma, sepsis and other immune deficient conditions in non-pregnant subjects [49]. Adipose tissue represents a major contributor to circulating TNF-α concentrations [50]. In the present study, we observed reduced plasma TNF-α concentrations in response to maternal taurine supplementation concomitant with reduced adiposity in the MOT mothers which is in agreement with previous observations whereby weight loss is associated with improvements in inflammatory markers [51].

We noted a reduction in maternal weight gain in taurine supplemented control and MO dams in the last stage of gestation. This was not reflected in an overall change in birth weights or a change in maternal water or food intake. There is evidence for a role of taurine in reducing fat mass in the rodent [52], [53] and in the present study we observed a reduction in fat mass in MOT dams but this was at the time of lactation; whether taurine had an effect on modifying maternal body composition in late pregnancy is not known.

Given the link between high fat diets and changes in glutamate metabolism [54], we also examine circulating glutamate levels in the lactating dams. Glutamate at high levels is well known to be toxic to the central nervous system [55], retinal neurons [56] and pancreatic islets [57]. As the fetal blood brain barrier is relatively permeable, even a slight increase in circulating glutamate has been shown to overstimulate neurons in the arcuate nucleus that can lead to metabolic dysregulation in later life [58]. Given the ability of glutamate to cross the placenta [59], it is possible that the elevated maternal glutamate levels observed in the present study may lead to significant adverse effects on later metabolic function in offspring. Taurine supplementation normalized circulating glutamate levels; previous studies have demonstrated that tissue specific levels of glutamate can be decreased by taurine supplementation. This may be due to taurine-mediated alterations in calcium influx via cysteine/glutamate antiport systems [60]–[62] and may be a mechanism by which beneficial effects of taurine are exerted in the present study but warrants further investigation. Of note in the present study was the sex-specific effects of maternal taurine on outcomes in the neonate. We have recently reported on sex-specific differences in placental weights in a model of maternal fructose intake [63] and it is possible that the sex-specific effects observed in offspring in the current study may in part be mediated by alterations in, for example, glutamate transfer across the placenta. In a recent paper by Tang *et al.* in the setting of a maternal low protein diet, it was shown that maternal taurine supplementation led to a reduction in insulin sensitivity in female but not male offspring although the mechanisms underpinning this sexual dimorphism are not clear [23].

Offspring hepatic inflammatory profile was significantly rescued as a result of maternal taurine supplementation. Data from the present study indicates that maternal obesogenic diet had adverse effects on neonatal hepatic inflammatory profiles. Increased expression of IL-1β and TNF-α receptors in MO offspring suggest that maternal developmental programming induces a predisposition to hepatic inflammatory responses which may contribute to long term risk of hepatic IR, steatosis and fibrosis. Notably, a recent study by Chiappini *et al*. demonstrated the importance of IL-1R1 overexpression in response to early development of obesity-induced NAFLD [64]. Furthermore, in other studies, IL1-R1 and IL-1β knockout mice display attenuated hepatic steatosis and inflammation when exposed to alcohol and high cholesterol diet [65], [66]. This suggests that reversal of IL1-R1 and TNFR1 in neonates from the MOT group may contribute to an overall improvement of long term metabolic health. We also examined parameters relating to IR and lipid metabolism (Figure S1); however these were unaffected in neonates which is unsurprising given the age and lack of metabolic challenge in these animals. Nevertheless, inflammation is a key pathological factor which we have shown can exacerbate hepatic steatosis. Therefore, the finding that maternal taurine supplementation reversed maternal diet-induced inflammatory receptor overexpression in neonates would suggest that taurine is having a protective effect on offspring liver inflammatory response and may confer protection against fatty liver disease in later life.

Additionally, we observed that in the control pregnancies, taurine supplementation increased neonatal mortality, despite no such an effect in the maternal obesogenic groups. Animal studies have demonstrated that taurine can prevent mortality in STD-induced diabetic adult rats [67]. However, there are limited data on possible adverse effects of taurine in normal pregnancies. Earlier work by Boujendar *et al.* reported adverse effects of taurine supplementation in offspring of control pregnancies as reflected in fetal hypoglycaemia and decreased pancreatic and postnatal body weights [21]. We did not observe any significant changes in neonatal glucose concentrations in the present study but there was a significant reduction in birthweight in female but not male offspring of taurine supplemented dams. Taurine supplementation *in vivo* has been reported to protect islets in offspring from low protein fed dams from cytokine toxicity, but increase islet sensitivity to cytokines and impair pancreatic development in control animals [21], [22]. Therefore, possible taurine toxicity in the setting of normal pregnancy outcomes should be further investigated.

In conclusion, a maternal obesogenic diet-induced postpartum impaired insulin sensitivity, hepatic steatosis and contributed to a programmed neonatal hepatic inflammatory profile. Maternal taurine supplementation exacerbated maternal hepatic steatosis yet benefited the circulating proinflammatory profile in dams and reversed the detrimental neonatal hepatic inflammatory cytokine receptor expression. Our findings suggest that maternal taurine supplementation may protect the offspring of obese mothers against developmentally programmed NAFLD despite worsening maternal postpartum fatty liver disease. While a few other studies investigated reversing the programming effects in offspring of undernutrition models [20]–[22], [68]–[70], our study first reports the reversing effect in a model of maternal obesity. Together, these studies indicate that the early period of life may be a critical window for reversing programming effects. However, while taurine supplementation during pregnancy may modify developmental programming of metabolic dysfunction in offspring, adverse maternal effects in normal pregnancies warrant caution and must be further investigated.

## Supporting Information

Figure S1 (TIF)

Table S1 Details of primers used in gene expression analysis.
(DOCX)

Table S2 Maternal gene expression main effects.
(DOCX)

## References

[1] Hillemeier MM, Weisman CS, Chuang C, Downs DS, McCall-Hosenfeld J, et al (2011) Transition to overweight or obesity among women of reproductive age. J Womens Health (Larchmt) 20: 703–710. 21599427 10.1089/jwh.2010.2397 PMC3096512

[2] Nodine PM, Hastings-Tolsma M (2012) Maternal obesity: improving pregnancy outcomes. MCN Am J Matern Child Nurs 37: 110–115. 22357072 10.1097/NMC.0b013e3182430296

[3] Metzger BE, Cho NH, Roston SM, Radvany R (1993) Prepregnancy weight and antepartum insulin secretion predict glucose tolerance five years after gestational diabetes mellitus. Diabetes Care 16: 1598–1605. 8299456 10.2337/diacare.16.12.1598

[4] Kaaja R, Laivuori H, Laakso M, Tikkanen MJ, Ylikorkala O (1999) Evidence of a state of increased insulin resistance in preeclampsia. Metabolism 48: 892–896. 10421232 10.1016/s0026-0495(99)90225-1

[5] Catalano PM (2010) Obesity, insulin resistance, and pregnancy outcome. Reproduction 140: 365–371. 20457594 10.1530/REP-10-0088 PMC4179873

[6] Tiikkainen M, Tamminen M, Hakkinen AM, Bergholm R, Vehkavaara S, et al (2002) Liver-fat accumulation and insulin resistance in obese women with previous gestational diabetes. Obes Res 10: 859–867. 12226133 10.1038/oby.2002.118

[7] Poston L, Harthoorn LF, Van Der Beek EM (2011) Obesity in pregnancy: implications for the mother and lifelong health of the child. A consensus statement. Pediatr Res 69: 175–180. 21076366 10.1203/PDR.0b013e3182055ede

[8] Gluckman PD, Hanson MA, Beedle AS (2007) Early life events and their consequences for later disease: a life history and evolutionary perspective. Am J Hum Biol 19: 1–19. 17160980 10.1002/ajhb.20590

[9] Fall CH, Osmond C, Barker DJ, Clark PM, Hales CN, et al (1995) Fetal and infant growth and cardiovascular risk factors in women. BMJ 310: 428–432. 7873947 10.1136/bmj.310.6977.428 PMC2548816

[10] Curhan GC, Chertow GM, Willett WC, Spiegelman D, Colditz GA, et al (1996) Birth weight and adult hypertension and obesity in women. Circulation 94: 1310–1315. 8822985 10.1161/01.cir.94.6.1310

[11] Samuelsson AM, Matthews PA, Argenton M, Christie MR, McConnell JM, et al (2008) Diet-induced obesity in female mice leads to offspring hyperphagia, adiposity, hypertension, and insulin resistance: a novel murine model of developmental programming. Hypertension 51: 383–392. 18086952 10.1161/HYPERTENSIONAHA.107.101477

[12] Howie GJ, Sloboda DM, Kamal T, Vickers MH (2009) Maternal nutritional history predicts obesity in adult offspring independent of postnatal diet. J Physiol 587: 905–915. 19103681 10.1113/jphysiol.2008.163477 PMC2669979

[13] Anuradha CV, Balakrishnan SD (1999) Taurine attenuates hypertension and improves insulin sensitivity in the fructose-fed rat, an animal model of insulin resistance. Can J Physiol Pharmacol 77: 749–754. 10588478

[14] Haber CA, Lam TK, Yu Z, Gupta N, Goh T, et al (2003) N-acetylcysteine and taurine prevent hyperglycemia-induced insulin resistance in vivo: possible role of oxidative stress. Am J Physiol Endocrinol Metab 285: E744–753. 12799318 10.1152/ajpendo.00355.2002

[15] Ito T, Schaffer SW, Azuma J (2012) The potential usefulness of taurine on diabetes mellitus and its complications. Amino Acids 42: 1529–1539. 21437784 10.1007/s00726-011-0883-5 PMC3325402

[16] Worden JA, Stipanuk MH (1985) A comparison by species, age and sex of cysteinesulfinate decarboxylase activity and taurine concentration in liver and brain of animals. Comp Biochem Physiol B 82: 233–239. 4053584 10.1016/0305-0491(85)90232-9

[17] Franconi F, Di Leo MA, Bennardini F, Ghirlanda G (2004) Is taurine beneficial in reducing risk factors for diabetes mellitus? Neurochem Res 29: 143–150. 14992273 10.1023/b:nere.0000010443.05899.2f

[18] Carneiro EM, Latorraca MQ, Araujo E, Beltra M, Oliveras MJ, et al (2009) Taurine supplementation modulates glucose homeostasis and islet function. J Nutr Biochem 20: 503–511. 18708284 10.1016/j.jnutbio.2008.05.008

[19] Nandhini AT, Thirunavukkarasu V, Anuradha CV (2005) Taurine modifies insulin signaling enzymes in the fructose-fed insulin resistant rats. Diabetes Metab 31: 337–344. 16369195 10.1016/s1262-3636(07)70202-1

[20] Cherif H, Reusens B, Ahn MT, Hoet JJ, Remacle C (1998) Effects of taurine on the insulin secretion of rat fetal islets from dams fed a low-protein diet. J Endocrinol 159: 341–348. 9795376 10.1677/joe.0.1590341

[21] Boujendar S, Reusens B, Merezak S, Ahn MT, Arany E, et al (2002) Taurine supplementation to a low protein diet during foetal and early postnatal life restores a normal proliferation and apoptosis of rat pancreatic islets. Diabetologia 45: 856–866. 12107730 10.1007/s00125-002-0833-6

[22] Merezak S, Reusens B, Renard A, Goosse K, Kalbe L, et al (2004) Effect of maternal low-protein diet and taurine on the vulnerability of adult Wistar rat islets to cytokines. Diabetologia 47: 669–675. 15298344 10.1007/s00125-004-1357-z

[23] Tang C, Marchand K, Lam L, Lux-Lantos V, Thyssen SM, et al (2013) Maternal taurine supplementation in rats partially prevents the adverse effects of early-life protein deprivation on beta-cell function and insulin sensitivity. Reproduction 145: 609–620. 23613616 10.1530/REP-12-0388

[24] Lin S, Hirai S, Yamaguchi Y, Goto T, Takahashi N, et al.. (2013) Taurine improves obesity-induced inflammatory responses and modulates the unbalanced phenotype of adipose tissue macrophages. Mol Nutr Food Res. 10.1002/mnfr.201300150 23939816

[25] Ainge H, Thompson C, Ozanne SE, Rooney KB (2011) A systematic review on animal models of maternal high fat feeding and offspring glycaemic control. Int J Obes (Lond) 35: 325–335. 20680016 10.1038/ijo.2010.149

[26] Matthews DR, Hosker JP, Rudenski AS, Naylor BA, Treacher DF, et al (1985) Homeostasis model assessment: insulin resistance and beta-cell function from fasting plasma glucose and insulin concentrations in man. Diabetologia 28: 412–419. 3899825 10.1007/BF00280883

[27] Livak KJ, Schmittgen TD (2001) Analysis of relative gene expression data using real-time quantitative PCR and the 2(-Delta Delta C(T)) Method. Methods 25: 402–408. 11846609 10.1006/meth.2001.1262

[28] Kleiner DE, Brunt EM, Van Natta M, Behling C, Contos MJ, et al (2005) Design and validation of a histological scoring system for nonalcoholic fatty liver disease. Hepatology 41: 1313–1321. 15915461 10.1002/hep.20701

[29] Stoeckman AK, Towle HC (2002) The role of SREBP-1c in nutritional regulation of lipogenic enzyme gene expression. J Biol Chem 277: 27029–27035. 12016216 10.1074/jbc.M202638200

[30] Horton JD, Shah NA, Warrington JA, Anderson NN, Park SW, et al (2003) Combined analysis of oligonucleotide microarray data from transgenic and knockout mice identifies direct SREBP target genes. Proc Natl Acad Sci U S A 100: 12027–12032. 14512514 10.1073/pnas.1534923100 PMC218707

[31] Foretz M, Pacot C, Dugail I, Lemarchand P, Guichard C, et al (1999) ADD1/SREBP-1c is required in the activation of hepatic lipogenic gene expression by glucose. Mol Cell Biol 19: 3760–3768. 10207099 10.1128/mcb.19.5.3760 PMC84202

[32] Postic C, Girard J (2008) Contribution of de novo fatty acid synthesis to hepatic steatosis and insulin resistance: lessons from genetically engineered mice. J Clin Invest 118: 829–838. 18317565 10.1172/JCI34275 PMC2254980

[33] Schoonjans K, Staels B, Auwerx J (1996) The peroxisome proliferator activated receptors (PPARS) and their effects on lipid metabolism and adipocyte differentiation. Biochim Biophys Acta 1302: 93–109. 8695669 10.1016/0005-2760(96)00066-5

[34] Steineger HH, Sorensen HN, Tugwood JD, Skrede S, Spydevold O, et al (1994) Dexamethasone and insulin demonstrate marked and opposite regulation of the steady-state mRNA level of the peroxisomal proliferator-activated receptor (PPAR) in hepatic cells. Hormonal modulation of fatty-acid-induced transcription. Eur J Biochem 225: 967–974. 7957233 10.1111/j.1432-1033.1994.0967b.x

[35] Hermanowski-Vosatka A, Gerhold D, Mundt SS, Loving VA, Lu M, et al (2000) PPARalpha agonists reduce 11beta-hydroxysteroid dehydrogenase type 1 in the liver. Biochem Biophys Res Commun 279: 330–336. 11118287 10.1006/bbrc.2000.3966

[36] Kim JK, Fillmore JJ, Chen Y, Yu C, Moore IK, et al (2001) Tissue-specific overexpression of lipoprotein lipase causes tissue-specific insulin resistance. Proc Natl Acad Sci U S A 98: 7522–7527. 11390966 10.1073/pnas.121164498 PMC34701

[37] Goldberg IJ, Eckel RH, Abumrad NA (2009) Regulation of fatty acid uptake into tissues: lipoprotein lipase- and CD36-mediated pathways. J Lipid Res 50 Suppl: S86–90 10.1194/jlr.R800085-JLR200 PMC2674753 19033209

[38] Havel PJ (2005) Dietary fructose: implications for dysregulation of energy homeostasis and lipid/carbohydrate metabolism. Nutr Rev 63: 133–157. 15971409 10.1301/nr.2005.may.133-157

[39] Ouyang X, Cirillo P, Sautin Y, McCall S, Bruchette JL, et al (2008) Fructose consumption as a risk factor for non-alcoholic fatty liver disease. J Hepatol 48: 993–999. 18395287 10.1016/j.jhep.2008.02.011 PMC2423467

[40] Chen SW, Chen YX, Shi J, Lin Y, Xie WF (2006) The restorative effect of taurine on experimental nonalcoholic steatohepatitis. Dig Dis Sci 51: 2225–2234. 17080243 10.1007/s10620-006-9359-y

[41] Chang YY, Chou CH, Chiu CH, Yang KT, Lin YL, et al (2011) Preventive effects of taurine on development of hepatic steatosis induced by a high-fat/cholesterol dietary habit. J Agric Food Chem 59: 450–457. 21126079 10.1021/jf103167u

[42] Gentile CL, Nivala AM, Gonzales JC, Pfaffenbach KT, Wang D, et al (2011) Experimental evidence for therapeutic potential of taurine in the treatment of nonalcoholic fatty liver disease. Am J Physiol Regul Integr Comp Physiol 301: R1710–1722. 21957160 10.1152/ajpregu.00677.2010 PMC3233850

[43] Bataller R, Brenner DA (2005) Liver fibrosis. J Clin Invest 115: 209–218. 15690074 10.1172/JCI24282 PMC546435

[44] Crespo J, Cayon A, Fernandez-Gil P, Hernandez-Guerra M, Mayorga M, et al (2001) Gene expression of tumor necrosis factor alpha and TNF-receptors, p55 and p75, in nonalcoholic steatohepatitis patients. Hepatology 34: 1158–1163. 11732005 10.1053/jhep.2001.29628

[45] Manco M, Marcellini M, Giannone G, Nobili V (2007) Correlation of serum TNF-alpha levels and histologic liver injury scores in pediatric nonalcoholic fatty liver disease. Am J Clin Pathol 127: 954–960. 17509993 10.1309/6VJ4DWGYDU0XYJ8Q

[46] Miura K, Kodama Y, Inokuchi S, Schnabl B, Aoyama T, et al.. (2010) Toll-like receptor 9 promotes steatohepatitis by induction of interleukin-1beta in mice. Gastroenterology 139: 323–334 e327. 10.1053/j.gastro.2010.03.052 PMC4631262 20347818

[47] Boden G, She P, Mozzoli M, Cheung P, Gumireddy K, et al (2005) Free fatty acids produce insulin resistance and activate the proinflammatory nuclear factor-kappaB pathway in rat liver. Diabetes 54: 3458–3465. 16306362 10.2337/diabetes.54.12.3458

[48] McArthur MJ, Atshaves BP, Frolov A, Foxworth WD, Kier AB, et al (1999) Cellular uptake and intracellular trafficking of long chain fatty acids. J Lipid Res 40: 1371–1383. 10428973

[49] Schuller-Levis GB, Park E (2003) Taurine: new implications for an old amino acid. FEMS Microbiol Lett 226: 195–202. 14553911 10.1016/S0378-1097(03)00611-6

[50] Ahima RS, Flier JS (2000) Adipose tissue as an endocrine organ. Trends Endocrinol Metab 11: 327–332. 10996528 10.1016/s1043-2760(00)00301-5

[51] Ziccardi P, Nappo F, Giugliano G, Esposito K, Marfella R, et al (2002) Reduction of inflammatory cytokine concentrations and improvement of endothelial functions in obese women after weight loss over one year. Circulation 105: 804–809. 11854119 10.1161/hc0702.104279

[52] Elshorbagy AK, Valdivia-Garcia M, Mattocks DA, Plummer JD, Orentreich DS, et al (2013) Effect of taurine and N-acetylcysteine on methionine restriction-mediated adiposity resistance. Metabolism 62: 509–517. 23154184 10.1016/j.metabol.2012.10.005

[53] Mikami N, Hosokawa M, Miyashita K (2012) Dietary combination of fish oil and taurine decreases fat accumulation and ameliorates blood glucose levels in type 2 diabetic/obese KK-A(y) mice. J Food Sci 77: H114–120. 22582992 10.1111/j.1750-3841.2012.02687.x

[54] Valladolid-Acebes I, Merino B, Principato A, Fole A, Barbas C, et al (2012) High-fat diets induce changes in hippocampal glutamate metabolism and neurotransmission. Am J Physiol Endocrinol Metab 302: E396–402. 22114023 10.1152/ajpendo.00343.2011

[55] Olney JW, Ho OL (1970) Brain damage in infant mice following oral intake of glutamate, aspartate or cysteine. Nature 227: 609–611. 10.1038/227609b0 5464249

[56] Kowluru RA, Engerman RL, Case GL, Kern TS (2001) Retinal glutamate in diabetes and effect of antioxidants. Neurochem Int 38: 385–390. 11222918 10.1016/s0197-0186(00)00112-1

[57] Davalli AM, Perego C, Folli FB (2012) The potential role of glutamate in the current diabetes epidemic. Acta Diabetol 49: 167–183. 22218826 10.1007/s00592-011-0364-z

[58] Hermanussen M, Garcia AP, Sunder M, Voigt M, Salazar V, et al (2006) Obesity, voracity, and short stature: the impact of glutamate on the regulation of appetite. Eur J Clin Nutr 60: 25–31. 16132059 10.1038/sj.ejcn.1602263

[59] Yu T, Zhao Y, Shi W, Ma R, Yu L (1997) Effects of maternal oral administration of monosodium glutamate at a late stage of pregnancy on developing mouse fetal brain. Brain Res 747: 195–206. 9045994 10.1016/s0006-8993(96)01181-x

[60] Bannai S (1986) Exchange of cystine and glutamate across plasma membrane of human fibroblasts. J Biol Chem 261: 2256–2263. 2868011

[61] Ientile R, Cangemi F, Di Giorgio RM, Macaione S (1992) Excess of taurine supplementation in rat: effects on GABA-related amino acids in developing nervous tissues. Ital J Biochem 41: 183–194. 1354213

[62] Yu X, Xu Z, Mi M, Xu H, Zhu J, et al (2008) Dietary taurine supplementation ameliorates diabetic retinopathy via anti-excitotoxicity of glutamate in streptozotocin-induced Sprague-Dawley rats. Neurochem Res 33: 500–507. 17762918 10.1007/s11064-007-9465-z

[63] Vickers MH, Clayton ZE, Yap C, Sloboda DM (2011) Maternal fructose intake during pregnancy and lactation alters placental growth and leads to sex-specific changes in fetal and neonatal endocrine function. Endocrinology 152: 1378–1387. 21303952 10.1210/en.2010-1093

[64] Chiappini F, Barrier A, Saffroy R, Domart MC, Dagues N, et al (2006) Exploration of global gene expression in human liver steatosis by high-density oligonucleotide microarray. Lab Invest 86: 154–165. 16344856 10.1038/labinvest.3700374

[65] Kamari Y, Shaish A, Vax E, Shemesh S, Kandel-Kfir M, et al (2011) Lack of interleukin-1alpha or interleukin-1beta inhibits transformation of steatosis to steatohepatitis and liver fibrosis in hypercholesterolemic mice. J Hepatol 55: 1086–1094. 21354232 10.1016/j.jhep.2011.01.048 PMC3210940

[66] Petrasek J, Bala S, Csak T, Lippai D, Kodys K, et al (2012) IL-1 receptor antagonist ameliorates inflammasome-dependent alcoholic steatohepatitis in mice. J Clin Invest 122: 3476–3489. 22945633 10.1172/JCI60777 PMC3461900

[67] Shivananjappa MM (2012) Muralidhara (2012) Taurine attenuates maternal and embryonic oxidative stress in a streptozotocin-diabetic rat model. Reprod Biomed Online 24: 558–566. 22414371 10.1016/j.rbmo.2012.01.016

[68] Vickers MH, Gluckman PD, Coveny AH, Hofman PL, Cutfield WS, et al (2005) Neonatal leptin treatment reverses developmental programming. Endocrinology 146: 4211–4216. 16020474 10.1210/en.2005-0581

[69] Vickers MH, Gluckman PD, Coveny AH, Hofman PL, Cutfield WS, et al (2008) The effect of neonatal leptin treatment on postnatal weight gain in male rats is dependent on maternal nutritional status during pregnancy. Endocrinology 149: 1906–1913. 18187552 10.1210/en.2007-0981

[70] Gray C, Li M, Reynolds CM, Vickers MH (2013) Pre-weaning growth hormone treatment reverses hypertension and endothelial dysfunction in adult male offspring of mothers undernourished during pregnancy. PLoS One 8: e53505. 23308239 10.1371/journal.pone.0053505 PMC3538633

