# Supplementary material for: Effects of Taurine Supplementation on Hepatic Markers of Inflammation and Lipid Metabolism in Mothers and Offspring in the Setting of Maternal Obesity
Source: PLoS One. 2013 Oct 17;8(10):e76961. doi: 10.1371/journal.pone.0076961 (PMC3798342; doi:10.1371/journal.pone.0076961)
Supplement: Table S1 — Details of primers used in gene expression analysis. (DOCX) [file pone.0076961.s002.docx]

**Supplementary Table 1. Details of primers used in gene expression analysis**

| Gene | Primer | Sequence/ Catalogue no./ Identification no. |
| --- | --- | --- |
| SREBP-1c | Self-designed by the NCBI online programme Primer BLAST against NM_001276707.1 | Fwd AGCCGTGGTGAGAAGCGCAC  Rev TGAGGGTGGAGGGGTCAGCG |
| PPARα | Self-designed by the NCBI online programme Primer BLAST against NM_013196.1 | Fwd GCAGAGGTCCGATTCTTCCA  Rev TCAGCATCCCGTCTTTGTTC |
| PEPCK | Self-designed by the NCBI online programme Primer BLAST against NM_198780.3 | Fwd AGGCTGGCTAAGGAGGAAGG  Rev ACCGTTTTCTGGGTTGATGG |
| LPL | QuantiTect Primer Assay (NM_012598) | QT00183218 |
| Fructokinase | QuantiTect Primer Assay (NM_031855) | QT00186305 |
| SIRT1 | QuantiTect Primer Assay (NM_001107627, XM_001080493, XM_228146) | QT02345854 |
| Cyclophilin A | Self-designed by the NCBI online programme Primer BLAST against NM_017101.1 | Fwd TTGGGTCGCGTCTGCTTCGA  Rev GCCAGGACCTGTATGCTTCA |
| β-actin | Self-designed by the NCBI online programme Primer BLAST against NM_031144.2 | Fwd CACCAACTGGGACGATATGGA  Rev CAGCCTGGATGGCTACGTACAT |
| FASN | TaqMan® Gene expression Assay Kit | Rn01463550_m1 |
| CD36 | TaqMan® Gene expression Assay Kit | Rn02115479_g1 |
| TNFα | TaqMan® Gene expression Assay Kit | Rn01525858_g1 |
| IL-1β | TaqMan® Gene expression Assay Kit | Rn00580432_m1 |
| IL-1R1 | TaqMan® Gene expression Assay Kit | Rn00565482_m1 |
| TNFR1 | TaqMan® Gene expression Assay Kit | Rn01492348_m1 |
| Cyclophilin A | TaqMan® Gene expression Assay Kit | Rn00690933_m1 |
| HPRT | TaqMan® Gene expression Assay Kit | Rn01527840_m1 |
