# Supplementary material for: Effects of Taurine Supplementation on Hepatic Markers of Inflammation and Lipid Metabolism in Mothers and Offspring in the Setting of Maternal Obesity
Source: PLoS One. 2013 Oct 17;8(10):e76961. doi: 10.1371/journal.pone.0076961 (PMC3798342; doi:10.1371/journal.pone.0076961)
Supplement: Table S2 — Maternal gene expression main effects. (DOCX) [file pone.0076961.s003.docx]

**Supplementary Table 2. Maternal gene expression main effects**

|  | Effect | | |
| --- | --- | --- | --- |
|  | Diet | Taurine | Interaction |
| SREBP1c | **F=6.737**  **P=0.015** | F=1.673  P=0.206 | F=0.484  P=0.492 |
| FASN | **F=24.352**  **P<0.001** | F=0.219  P=0.643 | F=2.898  P=0.100 |
| PPAR-α | **F=6.645**  **P=0.015** | F=1.272  P=0.269 | F=2.156  P=0.153 |
| LPL | **F=30.936**  **P<0.001** | F=0.578  P=0.454 | F=4.375  P=0.046 |
| Fructokinase | **F=27.354**  **P<0.001** | F=0.478  P=0.495 | F=7.012  P=0.013 |
| CD36 | F=0.410  P=0.527 | **F=11.700**  **P=0.002** | F=0.159  P=0.693 |
| SIRT1 | F=0.524  P=0.475 | F=0.137  P=0.714 | **F=6.450**  **P=0.017** |
| PEPCK | **F=44.324**  **P<0.001** | F=0.119  P=0.732 | F=0.995  P=0.327 |
| TNFα | F=15.385  P<0.001 | F=20.999  P<0.001 | **F=7.245**  **P=0.012** |
| IL1β | F=8.431  P=0.007 | F=15.040  P<0.001 | **F=4.626**  **P=0.040** |
| IL-1R1 | **F=5.073**  **P=0.032** | F=2.917  P=0.099 | F=2.510  P=0.124 |
| TNFR1 | F=1.008  P=0.324 | F=0.404  P=0.530 | F=0.616  P=0.439 |

Data represent an n=7-9 per group. SREPB1, sterol regulatory element-binding protein-1c; FASN, fatty acid synthase; PEPCK, phosphoenolpyruvate carboxykinase; LPL, lipoprotein lipase; PPAR-α, peroxisome proliferator-activated receptor alpha; SIRT1, silent mating type information regulation 2 homolog 1; CD36, cluster of differentiation 36; IL, interleukin,; TNF, tumor necrosis factor; R, receptor.
